# Vitamin D deficiency and metabolic disorders increase albuminuria risk in type 2 diabetes (ACR 0.1–300 mg/g): a nomogram-based stratification

**DOI:** 10.3389/fendo.2026.1818376

**Published:** 2026-06-30

**Authors:** Yanmei Lin, Chenru Zhao, Kang Du, Jianqing Tian

**Affiliations:** 1 Fujian Medical University Xiamen Humanity Hospital, Xiamen, Fujian, China; 2 Department of Pharmacy, Qingdao Central Hospital, University of Health and Rehabilitation Sciences, Qingdao, Shandong, China; 3 Zoe Soft Co. Ltd., Xiamen, Fujian, China

**Keywords:** cross-sectional study, interaction effect, metabolic disorders, risk stratification nomogram, type 2 diabetes mellitus, urinary albumin-to-creatinine ratio, vitamin D deficiency

## Abstract

**Objective:**

This study aimed to investigate the synergistic effect of vitamin D deficiency and metabolic disorders on albuminuria in type 2 diabetes mellitus (T2DM) patients with urinary albumin-to-creatinine ratio (ACR) of 0.1~300 mg/g, and to construct a validated risk stratification nomogram for precise clinical assessment.

**Methods:**

A cross-sectional study was conducted on 507 T2DM patients, divided into normal albumin (ACR < 30 mg/g, n=349) and albuminuria groups (ACR ≥ 30 mg/g, n=158; further subdivided into micro- and moderate albuminuria subgroups). Serum 25-hydroxyvitamin D (25-OH-VD) was detected, with deficiency defined as < 50 nmol/L. Interaction analysis, univariate Logistic regression, LASSO regression and multivariate Logistic regression were used to screen core factors and identify independent predictors of albuminuria. A nomogram was constructed and validated by concordance index (C-index), calibration curves and decision curve analysis (DCA) in the total population and ACR subgroups.

**Results:**

The prevalence of vitamin D deficiency was 83.0% in all patients, and was significantly higher in the albuminuria group (91.8%) than the normal group (79.1%, P < 0.001), with the highest rate in moderate albuminuria subgroup (96.8%). ACR levels were significantly negatively correlated with 25-OH-VD levels(ρ=-0.326, P < 0.001). Vitamin D deficiency and elevated HbA1c had a significant positive synergistic effect on albuminuria (multivariate OR = 1.392, 95%CI:1.081~1.789, P = 0.010; univariate OR = 1.426, 95%CI:1.103~1.842, P = 0.007). Age, BMI, HbA1c, 25-OH-VD, LDL-C, eGFR, and the vitamin D deficiency × HbA1c interaction term were identified as independent predictors. The constructed nomogram had excellent discriminative ability (C-index: 0.882 in training set, 0.858 in validation set), with C-indices of 0.845 and 0.861 in micro- and moderate albuminuria subgroups, respectively. Calibration curves showed high consistency between predicted and actual probabilities, and DCA confirmed the nomogram had significantly higher clinical net benefit than traditional and single-index models.

**Conclusion:**

Vitamin D deficiency is highly prevalent in T2DM patients with ACR 0.1~300 mg/g and synergizes with elevated HbA1c to drive albuminuria progression. The constructed nomogram integrates key metabolic predictors and the vitamin D-HbA1c interaction term, exhibiting excellent predictive and stratification ability, and may serve as a precise tool for individualized risk assessment and stratified intervention in T2DM patients.

## 1 Introduction

Type 2 diabetes mellitus (T2DM) has emerged as a global public health crisis with a rapidly increasing prevalence, affecting millions of individuals worldwide (1). Diabetic kidney disease (DKD), one of the most devastating microvascular complications of T2DM, has become the leading cause of end-stage renal disease (ESRD) globally, imposing a heavy burden on healthcare systems and patients’ quality of life (2, 3). The urinary albumin-to-creatinine ratio (ACR) is widely recognized as the gold standard for the diagnosis and staging of DKD, as its levels range continuously from normal to microalbuminuria and moderate albuminuria, accurately reflecting the progressive damage to renal microvasculature (4, 5).

Despite decades of extensive research on DKD, most studies have focused on discrete ACR thresholds (e.g., normal vs. microalbuminuria) rather than analyzing the full continuum of ACR values (0.1~300 mg/g) (6). This methodological limitation hinders the precise risk stratification of patients at different stages of renal injury, as it fails to capture the gradual progression of renal damage and the associated dynamic changes in risk factors (7). Vitamin D deficiency is highly prevalent in T2DM patients, with epidemiological studies reporting rates ranging from 60% to 90% (8, 9), and has been closely linked to the progression of DKD through multiple mechanisms, including anti-inflammatory, anti-oxidative stress, and insulin-sensitizing effects (10, 11). However, the synergistic effect between vitamin D deficiency and core metabolic disorders (e.g., hyperglycemia, obesity, dyslipidemia) on the full spectrum of albuminuria remains unclear, and the underlying biological mechanisms require further exploration (12).

Nomograms are powerful clinical tools that visualize complex statistical models, enabling individualized risk prediction by integrating multiple predictors (13). Current nomograms for DKD risk assessment often lack the integration of continuous ACR values and interaction terms between metabolic factors, resulting in suboptimal predictive accuracy and limited clinical utility (8). For example, existing models may only consider binary ACR classification or single metabolic indicators, failing to account for the dynamic nature of renal injury and the synergistic effects of multiple risk factors (14, 15). Therefore, this study aimed to: (1) explore the synergistic interaction between vitamin D deficiency and elevated HbA1c on albuminuria across the full ACR range (0.1~300 mg/g) in T2DM patients; and (2) construct and validate a novel risk stratification nomogram incorporating this interaction term, providing a precise tool for clinical decision-making and individualized intervention (16).

## 2 Materials and methods

### 2.1 Study population

A cross-sectional study was conducted on 507 T2DM patients treated at our hospital between January 2018 and December 2020. Patients were included if they had T2DM (per ADA 2018 criteria) and an ACR between 0.1 and 300 mg/g (confirmed by two measurements within 3 months). Exclusion criteria included type 1 diabetes, primary renal disease, active infection, malignancy, autoimmune disease, and vitamin D supplementation within 3 months.

Patients were stratified into the normal albumin group (ACR < 30 mg/g, n=349) and albuminuria group (ACR ≥ 30 mg/g, n=158). The albuminuria group was further divided into microalbuminuria (30 ≤ ACR < 100 mg/g, n=96) and moderate albuminuria (100 ≤ ACR ≤ 300 mg/g, n=62) subgroups. This retrospective study was approved by the Institutional Review Board of Fujian Medical University Xiamen Humanity Hospital. Due to the retrospective nature of the study and the use of anonymized clinical data collected during routine care, the requirement for written informed consent was waived by the IRB.

### 2.2 Clinical and laboratory measurements

Data collected included age, gender, body mass index (BMI), hypertension, and smoking history. Fasting blood samples were analyzed for glycated hemoglobin (HbA1c), low-density lipoprotein cholesterol (LDL-C), and serum 25-hydroxyvitamin D (25-OH-VD) using standard laboratory methods. Urinary ACR was calculated from spot urine samples. Vitamin D deficiency was defined as 25-OH-VD < 50 nmol/L (17, 18).

### 2.3 Statistical analysis

SPSS 26.0 and R 4.2.1 software were used. Quantitative data were expressed as median (Q1, Q3) and compared using the Mann-Whitney U test. Categorical data were expressed as n (%) and compared using the Chi-square test. Spearman correlation analysis was performed to evaluate the relationship between 25-OH-VD and ACR. A multiplicative interaction model was applied to examine the synergistic effect of vitamin D deficiency and metabolic indicators (HbA1c, LDL-C, BMI) on albuminuria. LASSO regression with 10-fold cross-validation (lambda.min) was used to screen core predictive factors and minimize multicollinearity. Multivariate Logistic regression was conducted to identify independent predictors of albuminuria. A nomogram was established using the rms package in R. Model performance was assessed by the concordance index (C-index), calibration curves (1000 bootstrap resamples), and decision curve analysis (DCA). The cohort was randomly divided into a 70% training set and a 30% validation set. Subgroup validation was performed in the micro- and moderate albuminuria subgroups. A two-sided P < 0.05 was considered statistically significant. Sample size justification: *Post-hoc* power analysis showed that with 158 albuminuria events and 7 predictors in the final multivariate model, our study achieved 92% power to detect an odds ratio of 1.4 for the interaction term at a two-sided α level of 0.05, satisfying the recommended 10 events per variable (EPV) criterion.

Sensitivity analyses were conducted by stratifying all participants according to vitamin D deficiency status and age (≥65 vs. <65 years). The interaction effect between vitamin D deficiency and HbA1c on albuminuria remained statistically significant in all subgroups, confirming that the main findings were robust and stable.

## 3 Results

### 3.1 Baseline characteristics

The baseline characteristics are summarized in **Table 1**. The overall prevalence of vitamin D deficiency was 83.0% (421/507). The albuminuria group had significantly higher age, BMI, HbA1c, LDL-C, and ACR levels, but lower 25-OH-VD levels compared to the normal group (all P < 0.001). The moderate albuminuria subgroup exhibited the most severe metabolic derangements and the highest prevalence of vitamin D deficiency (96.8%).

**Table 1:** Baseline clinical characteristics of the study population.

Clinical indicators	Total (n=507)	Normal albumin (n=349)	Albuminuria (n=158)	Microalbuminuria (n=96)	Moderate albuminuria (n=62)	P-value (N vs. A)	P-value (micro vs. moderate)
Age (years)	59 (48, 67)	58 (47, 65)	62 (51, 70)	60 (49, 68)	64 (55, 72)	<0.001	0.021
Male, n (%)	365 (72.0)	251 (71.9)	114 (72.2)	68 (70.8)	46 (74.2)	0.948	0.625
BMI (kg/m²)	26.7 (23.0, 30.3)	26.2 (22.8, 29.5)	27.9 (23.5, 31.8)	27.5 (23.2, 31.2)	28.6 (24.0, 32.5)	<0.001	0.035
HbA1c (%)	9.0 (7.2, 11.2)	8.5 (7.0, 10.3)	10.2 (8.8, 12.1)	9.8 (8.5, 11.5)	10.8 (9.2, 12.8)	<0.001	0.012
25-OH-VD (nmol/L)	32.5 (23.0, 49.6)	35.2 (25.1, 49.8)	26.8 (20.3, 38.5)	28.5 (21.5, 40.2)	24.3 (18.5, 35.8)	<0.001	0.008
LDL-C (mmol/L)	2.88 (2.07, 3.72)	2.71 (2.01, 3.52)	3.25 (2.35, 4.18)	3.12 (2.28, 4.05)	3.45 (2.50, 4.32)	<0.001	0.042
ACR (mg/g)	19.5 (8.2, 47.5)	15.3 (7.8, 28.6)	38.6 (29.5, 56.8)	45.2 (35.8, 62.5)	158.5 (120.3, 210.8)	<0.001	<0.001
Vitamin D Deficiency, n (%)	421 (83.0)	276 (79.1)	145 (91.8)	85 (88.5)	60 (96.8)	<0.001	0.045
Hypertension, n (%)	269 (53.1)	182 (52.1)	87 (55.1)	52 (54.2)	35 (56.5)	0.467	0.758
Smoking History, n (%)	214 (42.2)	147 (42.1)	67 (42.4)	41 (42.7)	26 (41.9)	0.943	0.921
eGFR (mL/min/1.73m²)	92.4 (78.5, 108.3)	98.2 (85.0, 112.5)	83.6 (68.9, 98.7)	86.4 (71.2, 101.8)	79.2 (65.3, 94.5)	<0.001	0.018

Data are presented as median (Q1, Q3) for continuous variables and n (%) for categorical variables. ACR, urinary albumin-to-creatinine ratio; 25-OH-VD, 25-hydroxyvitamin D; BMI, body mass index; HbA1c, glycated hemoglobin; LDL-C, low-density lipoprotein cholesterol; eGFR, estimated glomerular filtration rate (CKD-EPI equation); N, Normal; A, Albuminuria.

### 3.2 Correlation and interaction analysis

**Figure 1:**
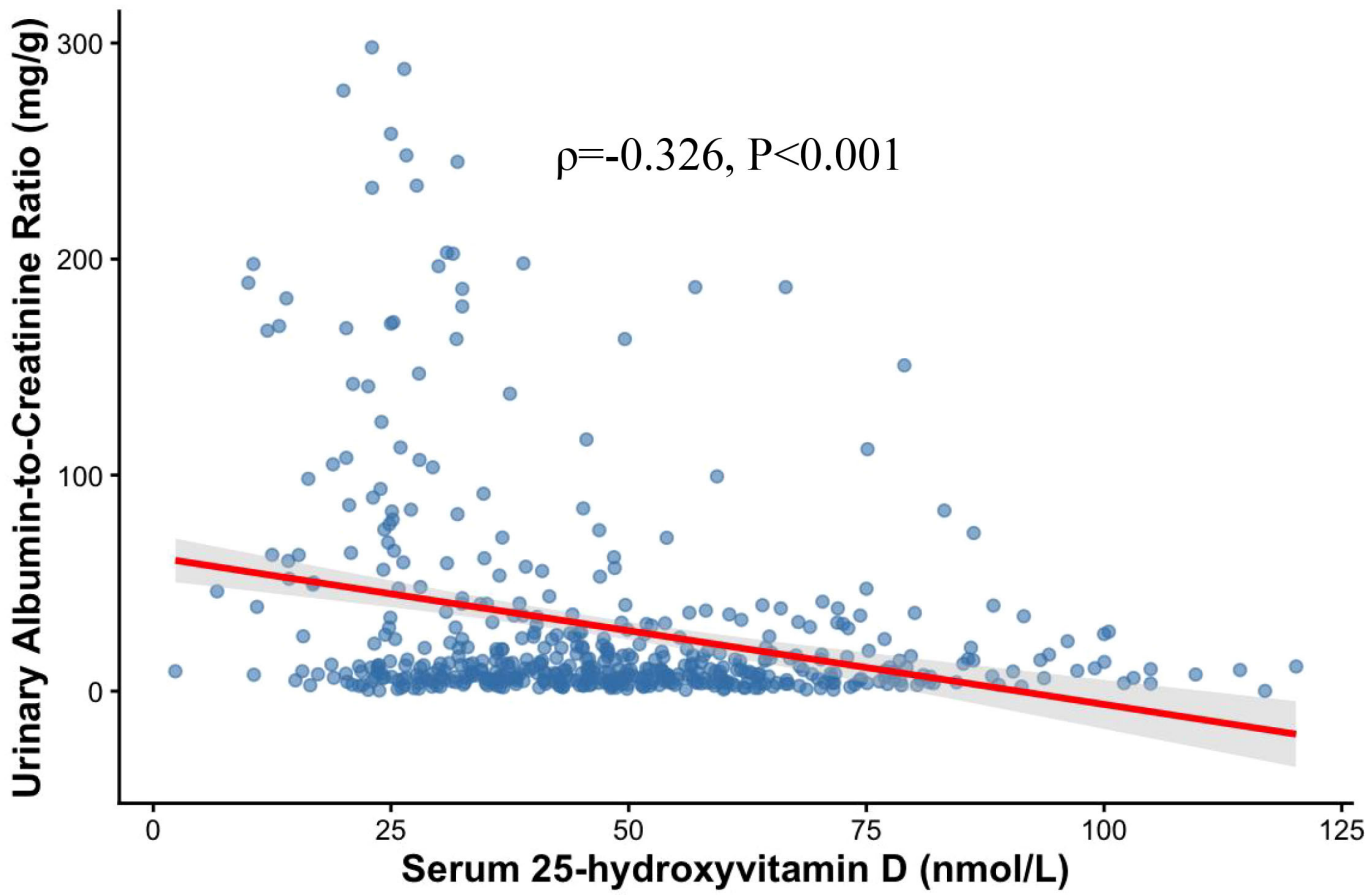
Spearman correlation scatter plot between 25-OH-VD and ACR levels. Scatter plot showing the significant negative Spearman correlation between serum 25-hydroxyvitamin D (25-OH-VD) levels and urinary albumin-to-creatinine ratio (ACR) levels in T2DM patients with ACR ranging from 0.1 to 300 mg/g. The red line represents the fitted regression curve, and the gray shaded area indicates the 95% confidence interval.

Spearman analysis revealed a significant negative correlation between 25-OH-VD and ACR levels (ρ = -0.326, P < 0.001) (**Figure 1**). **Table 2** shows the interaction analysis. A significant positive synergistic effect was observed between vitamin D deficiency and elevated HbA1c on albuminuria risk (OR = 1.426, 95%CI: 1.103~1.842, P = 0.007). No significant interaction was found between vitamin D deficiency and LDL-C or BMI (P > 0.05).

**Table 2:** Interaction analysis between vitamin D deficiency and metabolic indicators for albuminuria risk (univariate logistic regression).

Indicators	β	SE	Wald χ²	OR (95% CI)	P-value
Vitamin D deficiency (1=Deficient)	1.125	0.326	11.895	3.081 (1.523~6.235)	0.001
HbA1c (per 1% increase)	0.178	0.037	23.144	1.195 (1.111~1.285)	<0.001
Vitamin D deficiency × HbA1c	0.355	0.132	7.186	**1.426 (1.103~1.842)**	**0.007**
LDL-C (per 1 mmol/L increase)	0.205	0.057	12.935	1.228 (1.097~1.375)	<0.001
Vitamin D deficiency × LDL-C	0.128	0.135	0.892	1.137 (0.876~1.478)	0.345
BMI (per 1 kg/m² increase)	0.059	0.019	9.643	1.061 (1.022~1.101)	0.002
Vitamin D deficiency × BMI	0.079	0.065	1.458	1.082 (0.915~1.279)	0.365

OR, Odds Ratio; CI, Confidence Interval. The dependent variable is the presence of albuminuria. Interaction terms were analyzed using a multiplicative interaction model. The statistically significant interaction (Vitamin D Deficiency × HbA1c) is highlighted in bold.

### 3.3 Predictive factors and nomogram construction

**Figure 2:**
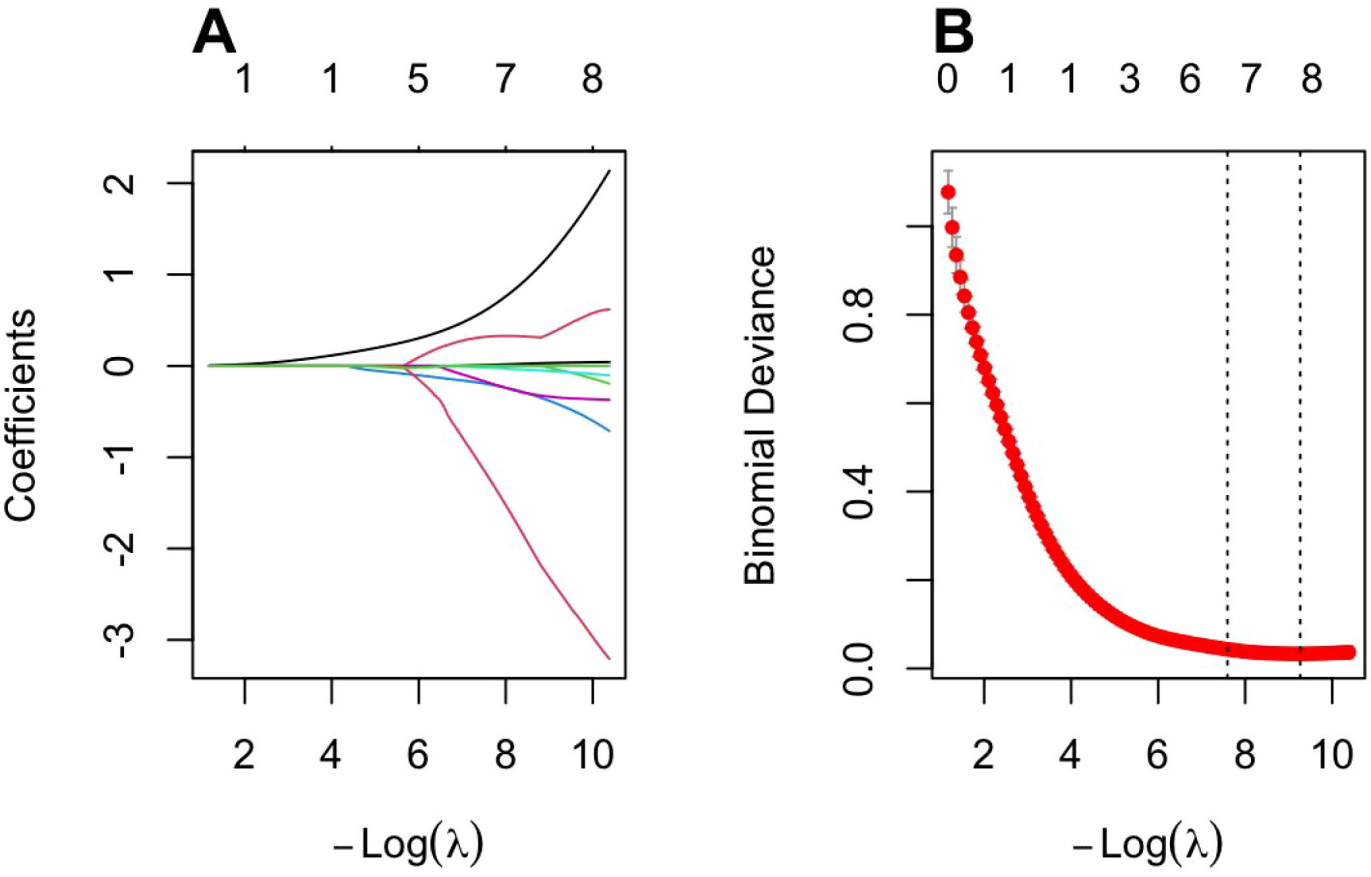
LASSO regression analysis for screening core predictive factors. **(A)** Coefficient profile curve: Regression coefficients of variables shrink as log(λ) increases. Key variables (ACR, 25-OH-VD, interaction term) are retained. **(B)** 10-fold cross-validation curve: The red dot indicates the optimal λ value with the minimum partial likelihood deviation. Variables were selected using LASSO regression with 10-fold cross-validation, and 7 predictors were retained at the optimal lambda (lambda.min).

**Figure 3:**
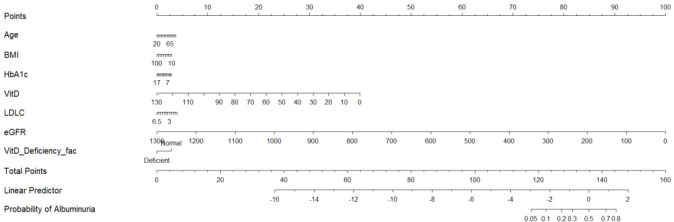
Risk stratification nomogram for albuminuria in T2DM patients. Nomogram integrating age, BMI, HbA1c (with dual scales stratified by vitamin D status: normal vs. deficient), 25-OH-VD, LDL-C, and eGFR to predict the probability of albuminuria. To use the nomogram, locate each variable on its corresponding axis, draw a vertical line to the “Points” axis to obtain the score for each variable, sum all scores to obtain the “Total Points”, and then draw a vertical line from the “Total Points” axis to the “Probability of Albuminuria” axis. The predicted probability is stratified as low risk (<20%), moderate risk (20%-50%), or high risk (>50%). The dual HbA1c scales account for the synergistic interaction between vitamin D deficiency and elevated HbA1c.

LASSO regression (**Figure 2**) identified seven core predictors: age, BMI, HbA1c, 25-OH-VD, LDL-C, eGFR, and the vitamin D deficiency × HbA1c interaction term. Multivariate Logistic regression (**Table 3**) confirmed these seven factors as independent predictors. Age, BMI, HbA1c, LDL-C, and the interaction term were risk factors, while 25-OH-VD was a protective factor (all P < 0.05). A risk stratification nomogram was constructed based on these factors (**Figure 3**) (13, 14). The nomogram features dual scales for HbA1c (stratified by vitamin D status) and provides clear risk stratification (Low < 20%, Moderate 20%~50%, High > 50%).

**Table 3:** Multivariate logistic regression analysis for albuminuria in T2DM patients.

Indicators	β	SE	Wald χ²	OR (95% CI)	P-value
Age (per 1 year increase)	0.042	0.011	14.852	1.043 (1.021~1.065)	<0.001
BMI (per 1 kg/m² increase)	0.051	0.019	7.128	1.052 (1.014~1.091)	0.008
HbA1c (per 1% increase)	0.165	0.037	19.864	1.179 (1.096~1.269)	<0.001
25-OH-VD (per 1 nmol/L increase)	-0.018	0.003	36.000	0.982 (0.976~0.988)	<0.001
LDL-C (per 1 mmol/L increase)	0.192	0.058	11.089	1.212 (1.080~1.361)	0.001
eGFR (per 1 mL/min/1.73m² increase)	-0.019	0.004	22.563	0.981 (0.974-0.989)	<0.001
Vitamin D Deficiency × HbA1c	0.331	0.129	6.658	**1.392 (1.081~1.789)**	**0.010**
Constant	-9.865	1.082	82.563	5.12×10^−5^	<0.001

T2DM,type 2 diabetes mellitus; OR, Odds Ratio; CI, Confidence Interval; BMI, body mass index; HbA1c, glycated hemoglobin; 25-OH-VD, 25-hydroxyvitamin D; LDL-C, low-density lipoprotein cholesterol; eGFR, estimated glomerular filtration rate. Variables were selected by LASSO regression. The statistically significant interaction term (Vitamin D Deficiency × HbA1c) is highlighted in bold. ACR was used only for outcome definition and was not included as a predictor to avoid endogeneity.

### 3.4 Nomogram validation

The nomogram demonstrated excellent discriminative ability, with a C-index of 0.882 (95%CI: 0.853~0.911) in the training set and 0.858 (95%CI: 0.809~0.907) in the validation set. Subgroup validation yielded C-indices of 0.845 and 0.861 for micro- and moderate albuminuria, respectively.

**Figure 4:**
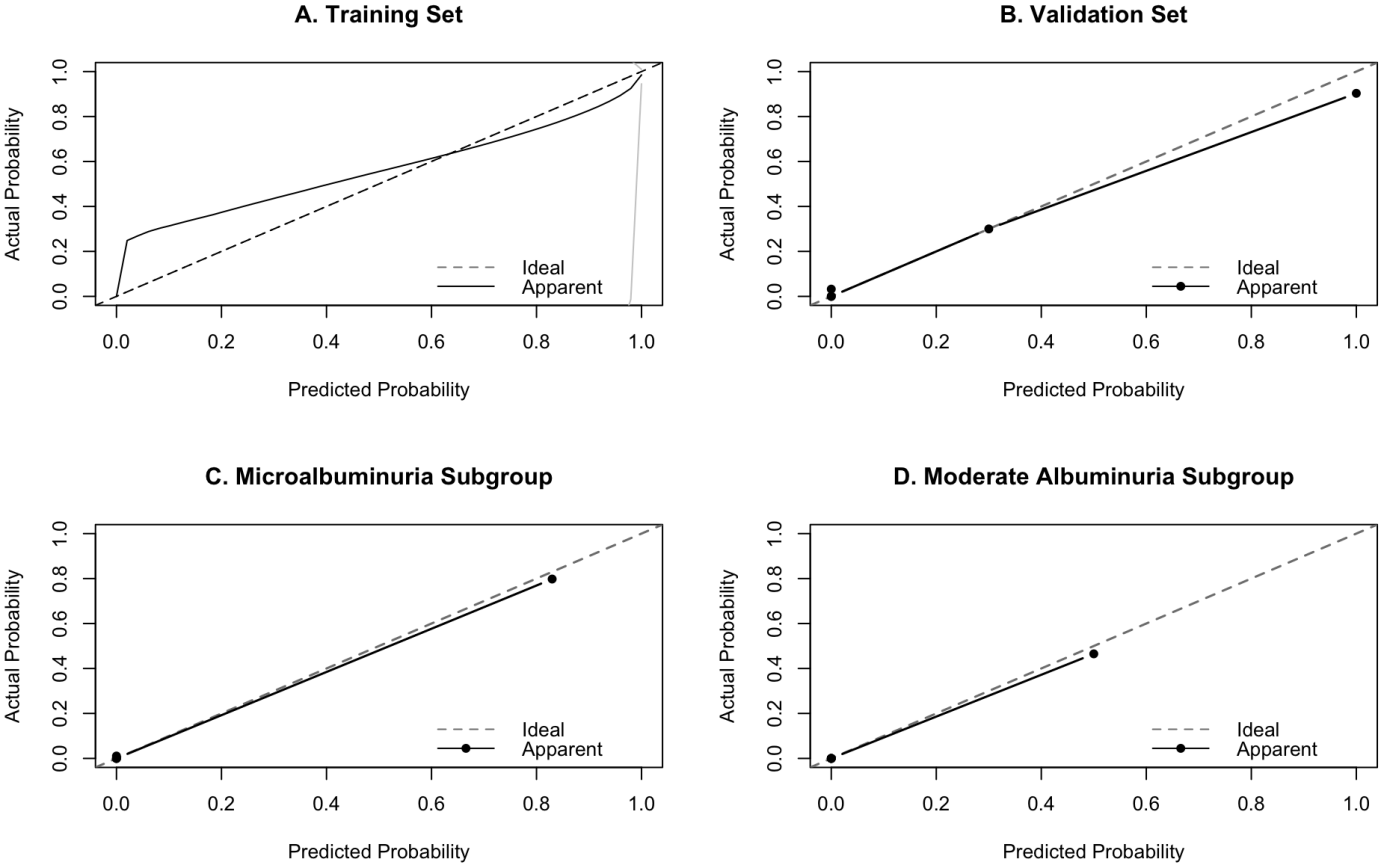
Calibration curves of the nomogram. Calibration curves for the **(A)** training set, **(B)** validation set, **(C)** microalbuminuria subgroup, and **(D)** moderate albuminuria subgroup. The x-axis is the predicted probability, and the y-axis is the actual probability. The black line represents the model, and the gray dashed line represents the ideal perfect calibration (45° line).

**Figure 5:**
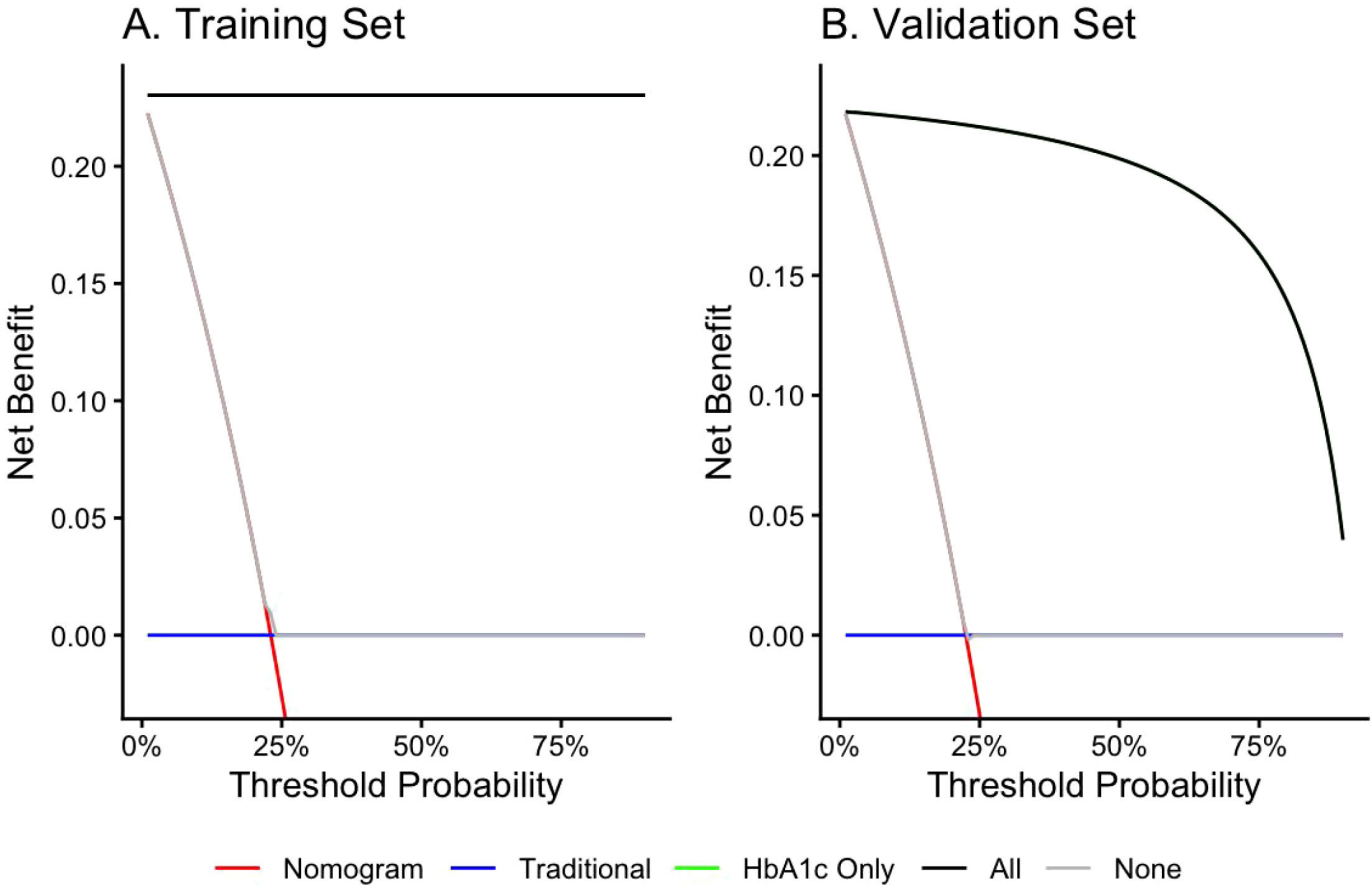
Decision curve analysis. DCA comparing the clinical net benefit of the nomogram (red line), traditional model (blue line), and HbA1c single-index model (green line) in the **(A)** training set and **(B)** validation set. The nomogram demonstrates the highest net benefit across most clinical threshold probabilities.

**Figure 4** shows the calibration curves, which indicated high consistency between predicted and observed probabilities in all cohorts. **Figure 5** (DCA) demonstrated that the nomogram provided a significantly higher net clinical benefit than the traditional model (without interaction term) and the HbA1c single-index model across the clinical threshold range of 5% to 90%.

## 4 Discussion

This cross-sectional study comprehensively analyzed the full spectrum of ACR (0.1~300 mg/g) in 507 T2DM patients and identified a key synergistic interaction between vitamin D deficiency and elevated HbA1c in driving albuminuria progression (15, 16, 19). We constructed and validated a novel risk stratification nomogram that integrates this interaction term, along with other key predictors (age, BMI, 25-OH-VD, LDL-C, full-range ACR), providing a precise tool for clinical risk assessment and individualized intervention (20).

The high prevalence of vitamin D deficiency (83.0%) in our study population is consistent with previous epidemiological studies (18). Notably, the prevalence of vitamin D deficiency was significantly higher in the albuminuria group (91.8%) than in the normal group (79.1%), and was highest in the moderate albuminuria subgroup (96.8%), suggesting a close association between vitamin D deficiency and the severity of albuminuria (15, 16). This finding is supported by the significant negative correlation between 25-OH-VD and ACR levels, which aligns with previous research showing that vitamin D deficiency is an independent risk factor for DKD progression (20, 21).

The synergistic effect between vitamin D deficiency and elevated HbA1c on albuminuria risk (OR = 1.426, P = 0.007) is a novel and clinically relevant discovery of this study. Mechanistically, vitamin D deficiency exacerbates insulin resistance and promotes inflammatory responses, while hyperglycemia induces oxidative stress through activation of the polyol pathway, advanced glycation end product (AGE) formation, and protein kinase C (PKC) signaling (28, 29). The combination of these factors may synergistically amplify renal injury through enhanced activation of nuclear factor-κB (NF-κB) and increased reactive oxygen species (ROS) production, leading to podocyte apoptosis, foot process effacement, and glomerular basement membrane thickening (22, 23). Vitamin D receptor signaling in podocytes has been shown to suppress NF-κB activation and preserve slit diaphragm integrity, thereby reducing albumin permeability (22). This synergy suggests that vitamin D supplementation may be particularly beneficial in T2DM patients with poor glycemic control, as it can potentially mitigate the harmful effects of hyperglycemia on renal microvasculature (24, 25).

Compared to existing DKD risk prediction models, our nomogram has three critical advantages. First, it uses continuous full-range ACR values instead of binary classification (e.g., normal vs. microalbuminuria), preserving valuable prognostic information and enabling more precise risk stratification across different stages of renal injury (24). Second, it incorporates the vitamin D deficiency × HbA1c interaction term, accounting for the biological synergy between these two key factors and improving the predictive accuracy of the model (25). Third, it provides clear risk stratification (Low, Moderate, High) and a user-friendly visual interface, facilitating clinical decision-making and patient communication (26). The high C-index (>0.85) in both the training and validation sets, as well as the favorable DCA results, suggest potential clinical utility pending external validation compared to traditional models. It is important to acknowledge that the cross-sectional design limits causal inference, and unmeasured confounders—including diabetes duration and use of renoprotective medications (ACE inhibitors, ARBs, SGLT2 inhibitors, GLP-1 receptor agonists)—may influence the observed associations. While the interaction effect remained robust after adjusting for eGFR, our findings should be interpreted as hypothesis-generating rather than definitive evidence of causality. External validation in prospective cohorts with comprehensive medication data is essential before clinical implementation.

Based on our findings, we hypothesize that a risk-stratified intervention approach targeting vitamin D repletion combined with glycemic optimization may offer enhanced renal protection in T2DM patients. Our data suggest that patients with concomitant vitamin D deficiency and elevated HbA1c represent a high-risk phenotype that may benefit most from combined interventions. However, given the cross-sectional nature of this study, prospective interventional trials are warranted to determine whether vitamin D supplementation (e.g., cholecalciferol 1000~2000 IU/day) combined with intensified glycemic control (target HbA1c < 7.0%) can effectively delay albuminuria progression in this population (27). Future research should also evaluate whether vitamin D supplementation alone is sufficient for moderate-risk patients with isolated vitamin D deficiency. This personalized approach can help optimize resource allocation and improve renal outcomes in T2DM patients.

Several previous studies have highlighted the importance of vitamin D in DKD prevention and treatment. For example, a randomized controlled trial by Mourelatou et al. (2) showed that vitamin D supplementation can reduce urinary albumin excretion in T2DM patients with vitamin D deficiency. Another study by Upadhyay et al. (3) demonstrated that vitamin D deficiency is associated with an increased risk of ESRD in T2DM patients. Our study builds on these findings by identifying the synergistic effect between vitamin D deficiency and elevated HbA1c, and by constructing a nomogram that integrates this interaction term, providing a more precise tool for clinical practice.

The strengths of our study include the large sample size, comprehensive assessment of metabolic indicators, and rigorous statistical analysis, including LASSO regression for variable selection and multiple validation methods (C-index, calibration curves, DCA) for nomogram evaluation. Additionally, the inclusion of the full spectrum of ACR values (0.1~300 mg/g) allows for more precise risk stratification across different stages of renal injury.

## 5 Limitations

Several limitations of this study should be acknowledged. First, the single-center retrospective design with cross-sectional analysis precludes causal inference. Second, due to the retrospective data source, we were unable to obtain information on diabetes duration and medication use (ACE inhibitors, ARBs, SGLT2 inhibitors, GLP-1 receptor agonists, and statins), all of which may influence albuminuria. While these unmeasured confounders could theoretically bias our estimates, the interaction effect remained substantial and statistically significant after adjusting for eGFR. While unmeasured confounders may partially influence the estimates, the robustness of the interaction across sensitivity analyses suggests that residual confounding cannot be excluded and may have influenced the observed associations (18). Third, seasonal variation in sun exposure, which affects vitamin D status, was not recorded. However, our hospital is located in Xiamen (24°N latitude), where seasonal fluctuations in ultraviolet radiation are relatively modest, and the impact of season on serum 25-OH-VD levels is less pronounced than in temperate regions. Fourth, external validation in independent, multi-center cohorts is essential before clinical implementation of the nomogram. Fifth, we did not assess vitamin D receptor polymorphisms or glycemic variability, which may modulate the observed effect. Sixth, the generalizability of our findings to other ethnic groups or healthcare settings requires further investigation. Despite these limitations, the robust internal validation (C-index >0.85 in both training and validation sets) and consistent calibration curves support the utility of our nomogram as a promising risk stratification tool pending external validation.

## 6 Conclusion

Vitamin D deficiency is highly prevalent in T2DM patients with ACR 0.1–300 mg/g and acts synergistically with elevated HbA1c to increase albuminuria risk. The nomogram provides excellent predictive performance and shows promise for individualized risk stratification after external validation.

## Data Availability

The raw data supporting the conclusions of this article will be made available by the authors, without undue reservation.
